# Supplementary material for: Appropriate Method of Administering Vasopressors for Maternal Hypotension Associated with Combined Spinal Epidural Anesthesia in Elective Cesarean Section: Impact on Postnatal Respiratory Support for Newborns
Source: Medicina (Kaunas). 2022 Mar 8;58(3):403. doi: 10.3390/medicina58030403 (PMC8950217; doi:10.3390/medicina58030403)
Supplement: Supplementary file 1 [file medicina-58-00403-s001.zip › medicina-1586433-supplementary.pdf]

Supplementary Table S1

Table S1. The results of correlation between blood pressure-related parameters.

		**maxSBP**	**miniSBP**	**difference of SBP**	**maxDBP**	**miniDBP**	**difference of DBP**
maxSBP	coefficient	1	0.087	0.736**	0.652**	-0.035	0.512**
	p		0.200	<0.01	<0.01	0.611	<0.01
	number	220	220	220	220	220	220
miniSBP	coefficient	0.087	1	-0.610**	0.167*	0.716**	-0.460**
	p	0.200		<0.01	0.013	<0.01	<0.01
	number	220	220	220	220	220	220
difference of SBP	coefficient	0.736**	-0.610**	1	0.405**	-0.513**	0.719**
	p	<0.01	<0.01		<0.01	<0.01	<0.01
	number	220	220	220	220	220	220
maxDBP	coefficient	0.652**	0.167*	0.405**	1	0.179**	0.596**
	p	<0.01	0.013	<0.01		<0.01	<0.01
	number	220	220	220	220	220	220
miniDBP	coefficient	-0.035	0.716**	-0.513**	0.179**	1	-0.683**
	p	0.611	<0.01	<0.01	<0.01		<0.01
	number	220	220	220	220	220	220
difference of DBP	coefficient	0.512**	-0.460**	0.719**	0.596**	-0.683**	1
	p	<0.01	<0.01	<0.01	<0.01	<0.01	
	number	220	220	220	220	220	220

**： $p < 0.05$, *： $p < 0.01$
